# EU CTR 536/2014: Are We Able to Reap the Benefits by Now?

**DOI:** 10.34172/aim.30011

**Published:** 2024-05-06

**Authors:** Venu Gopal Jonnalagadda

**Affiliations:** ^1^Bugworks Research India Pvt. Ltd., Centre for Cellular & Molecular Platforms (C-CAMP), National Centre for Biological Sciences (NCBS), GKVK Campus, Tata Institute of Fundamental Research, Bangalore, India

 As we enter 2024, it is necessary to look back on the major EU clinical trial regulation (EU CTR) after implementation from 31 January, 2022 to understand the major overhaul in the clinical trials arena.^1^

 To begin with, this regulation was designed to attract the sponsors to be perceived as easier when compared with other geographical regions and to increase the transparency alongside with regards to documentation, location, and status of the trial by introducing the new clinical trial information system (CTIS) platform.^2^

 Secondly, although, EU CTR was implemented to establish a harmonious environment, it does face the challenges of variation, where the regulation is “silent” on various documents of the clinical trial application, i.e. protocol synopsis, curriculum vitae (CV) of the principal investigator (PI), and patient facing documents, in addition to the translated documents.^3^

 Thirdly, request for information (RFI) from the regulatory authorities forms a major critical part of the process. The duration of 12 days is short and it will jeopardize the activities, quality and turnaround from the parties involved in this response, such as a contract research organization (CRO), translation vendors, and so on.^4^

 Fourthly, the European Medicines Agency (EMA) organized a consultation with all stakeholders in May 2023 and in response to it, the revised rules were published in October 2023 on protecting personal and commercially confidential information while ensuring that information relevant to patients and researchers is made available.^4^

 As we understand, any change to the system will start with denial, followed by anger, exploring and acceptance. Finally, keeping abreast of new information with changes, providing guidance to the sponsors, investigators, patients, stakeholders will a make a significant change to the clinical trials domain to deliver the improvements in efficiency, quality, and timeliness which requires patience and persistence.

 When things look dark, there is always a “silver lining”.
